# Comparison of Endoscopic and Microscopic Surgery for the Treatment of Acquired Cholesteatoma by EAONO/JOS Staging

**DOI:** 10.3390/healthcare12171737

**Published:** 2024-08-31

**Authors:** Ayaka Otsuka, Hajime Koyama, Akinori Kashio, Yu Matsumoto, Tatsuya Yamasoba

**Affiliations:** 1 Department of Otorhinolaryngology, Mitsui Memorial Hospital, Tokyo 101-8643, Japan; otsuka-ayaka@mitsuihosp.or.jp; 2 Department of Otorhinolaryngology and Head and Neck Surgery, The University of Tokyo, Tokyo 113-8655, Japan; kashioa-tky@umin.ac.jp; 3 Department of Otorhinolaryngology, Tokyo Metropolitan Police Hospital, Tokyo 164-8541, Japan; y.matsumoto@keisatsubyoin.or.jp; 4 Department of Otorhinolaryngology, Tokyo Teishin Hospital, Tokyo 102-8798, Japan

**Keywords:** endoscopic surgery, microscopic surgery, cholesteatoma, EAONO/JOS staging

## Abstract

Cholesteatoma is a benign tumor of the middle ear. Tympanoplasty is performed to remove cholesteatoma, prevent recurrence, and avoid complications. Previously, microscopy was used for tympanoplasty; however, endoscopy has become increasingly popular for this procedure. The effectiveness of endoscopy and the appropriate candidacy for endoscopic ear surgery remain controversial. In this retrospective chart review study, we enrolled 107 patients with cholesteatoma who underwent tympanoplasty and compared the microscopic approach (71 patients) and the endoscopic approach (36 patients) at different stages to clarify the benefits of using endoscopy and to determine candidacy for endoscopic ear surgery. Approach selection, complication rate, recurrence rate, and postoperative hearing threshold were compared between microscopic and endoscopic approaches in stages I, II, and III. Endoscopy was used more often than microscopy for early-stage (stage I) cholesteatoma (*p* = 0.005) and less frequently for advanced-stage (stage II) cholesteatoma (*p* = 0.02). Endoscopy surgery resulted in chorda tympani injury less often than microscopic surgery (*p* = 0.049); however, there were no significant differences between these two groups in terms of recurrence rate or postoperative hearing outcome. Endoscopy is particularly useful for early-stage cholesteatoma, and both approaches show no difference in hearing outcome in stage I and II; nevertheless, further research is required to determine an appropriate approach for more advanced stages (stage III).

## 1. Introduction

Acquired cholesteatoma is a benign tumor consisting of keratinizing squamous epithelium [1]. Expansion of a cholesteatoma can erode surrounding bony structures, causing complications such as otorrhea, hearing loss, dizziness, as well as facial paralysis and intracranial complications [2]. The surgical intervention for cholesteatoma aims to eradicate the lesion with the best procedures to prevent recurrence. Traditionally, the microscopic approach has been used to treat this disease. Microscopic ear surgery (MES) provides surgeons with binocular vision, depth perception, and the ability to perform two-handed surgery, which could be beneficial for surgical management. However, the microscope provides a limited view of hidden areas, such as the anterior epitympanic recess or the tympanic sinus [3]. To overcome these disadvantages, endoscopic surgery has recently been introduced.

Endoscopic evaluation of the tympanic cavity was first proposed in the 1980s, and endoscopic ear surgery (EES) was first performed in the 1990s. Recent development of endoscopic technology makes EES easier to perform [4,5]. The endoscope provides a wider and clearer view with optimal magnification, which could reduce the residual lesions. Moreover, the endoscopic transcanal approach follows the rational route of cholesteatoma growth, increasing the possibility of completely removing cholesteatoma while preserving intact ossicles.

Many studies and some reviews have reported the effectiveness of EES for the treatment of cholesteatoma [6,7]. They demonstrated that EES could reduce the recurrence rate and shorten the operation time, with no significant difference in postoperative hearing outcomes compared with MES [8,9,10,11]. However, cholesteatoma presents diverse conditions, necessitating consideration of stage differences.

The EAONO/JOS joint consensus statements are the first classification of cholesteatoma [12]. They divide the middle ear space and stratify cholesteatoma based on location and extracranial complications or pathologic conditions. This classification aims to establish a global standard for comparing postoperative outcomes in both MES and EES [13,14]. However, despite our research efforts, there is no direct comparison of complications, recurrence rates, or hearing outcomes for cholesteatoma at each stage between EES and MES. Therefore, the difference between MES and EES at the same stage remains unknown.

In this study, using the EAONO/JOS joint consensus statements, we aimed to compare MES and EES in treating cholesteatoma, considering the division of stages, and to clarify the effectiveness of these approaches across different stages.

## 2. Materials and Methods

### 2.1. Study Designs

**Figure 1:**
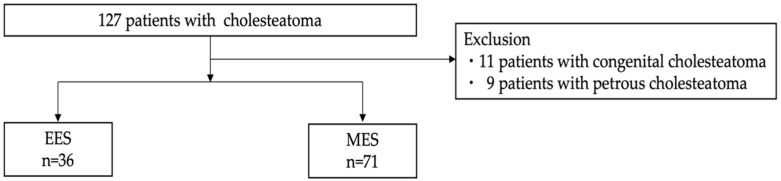
Among the 127 patients with cholesteatoma, 20 patients with congenital cholesteatoma or petrous cholesteatoma were excluded, and 36 patients received EES and 71 patients received MES. EES, endoscopic ear surgery; MES, microscopic ear surgery.

Data from 127 patients with cholesteatoma, who were treated at the Department of Otolaryngology and Head and Neck Surgery of The University of Tokyo Hospital between January 2017 and March 2021, were included in this study. Eleven patients with congenital cholesteatoma and nine patients with petrous cholesteatoma were excluded. The follow-up period lasted until March 2022. Using retrospective chart review, patients were divided into two groups based on whether they received EES (36 patients) or MES (71 patients) (Figure 1). We used tympanic membrane findings, audiometry, temporal bone CT, and in some cases MRI, which has been reported to be useful in determining the exact location of cholesteatoma [15], to identify the lesion and perform preoperative planning, allowing surgeons to choose the most appropriate surgical approach. All surgeries were performed by experienced otologists in our hospital, with the choice of EES or MES determined by individual otologists’ judgments.

We classified cholesteatoma into the following categories based on disease type:

- Pars flaccida
- Pars tensa
- Secondary
- Combination
- Unclassifiable

Additionally, we staged cholesteatoma according to the extension of the disease using the EAONO/JOS classification:

- Stage I: Cholesteatoma localized in the primary site.
- Stage II: Cholesteatoma involving two or more sites.
- Stage III: Cholesteatoma with extracranial complications and/or intratemporal pathological conditions.
- Stage IV: Cholesteatoma with intracranial complications.

**Figure 2:**
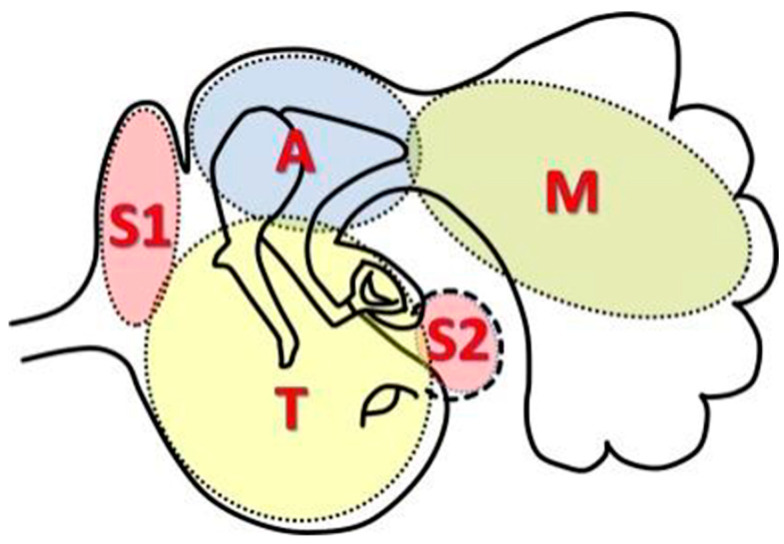
Divisions of the middle ear space (STAM system). Based on the EAONO/JOS system 2017 classification and staging of middle ear cholesteatoma, in the area of cholesteatoma, the tympanomastoid space was divided into four sections: the difficult-access sites (S), the tympanic cavity (T), the attic (A), and the mastoid (M). The difficult-access sites (S) included S1, the supratubal recess (also called the anterior epitympanum or protympanum) and S2, the sinus tympani. The posterior border of the attic was the posterior end of the incus short process or the fossa incudis. The mastoid included the antrum and the mastoid cells.

In the region of cholesteatoma, the tympanomastoid space was divided into four sections (Figure 2 [12]): 

S: Difficult-access sites, including S1 (the supratubal recess, also known as the anterior epitympanum or protympanum) and S2 (the sinus tympani).

T: Tympanic cavity.

A: Attic (with the posterior border defined by the posterior end of the incus short process or the fossa incudis).

M: Mastoid (including the antrum and the mastoid cells).

### 2.2. Outcome Measures 

Types and stages of cholesteatoma were classified in the EES and MES groups. Types of cholesteatoma were classified into pars flaccida, pars tensa, secondary, combination, or unclassifiable. Stages of cholesteatoma were classified into stage I, stage II, stage III, or stage IV.

The main outcome measures were complications, recurrence rate of cholesteatoma after initial surgery in our hospital, and postoperative hearing thresholds. We compared the main outcomes by stage of cholesteatoma between the EES group and the MES group.

Audiometric data included preoperative and postoperative air conduction threshold averages at four tested frequencies (500, 1000, 2000, and 4000 Hz). Postoperative outcomes were calculated from the hearing assessments one year after surgery. 

Complications were evaluated during and after surgery. Injury to the tympanic nerve was assessed by whether it was severed intraoperatively, and postoperative hematoma was evaluated by inspection and palpation [16].

### 2.3. Statistical Analysis

To compare the EES group with MES group, statistical analysis was performed using the chi-squared test or Fisher’s exact test for cholesteatoma types and stages (BellCurve for Excel version 4.04). Welch’s *t*-test was used to compare postoperative hearing thresholds between the two groups. Statistical significance was set at *p* < 0.05.

## 3. Results

Thirty-six patients (17 female, 19 male) underwent EES, while 71 patients (18 female, 53 male) underwent MES. In the EES group, one case required a second stage because it was difficult to completely remove the cholesteatoma matrix that was adhered around the stapes. In the MES group, nine cases were treated as second-stage because cholesteatoma had adhered around the stapes or had developed into the anterior tympanic cavity or tympanic sinus, which made it difficult to confirm the complete removal. Four patients who underwent a dual approach were included in the MES group. The mean age ± standard deviation (SD) was 46 ± 20.7 years in the EES group and 44 ± 20.4 years in the MES group. 

### 3.1. Types of Cholesteatoma (Figure 3, Table 1)

Pars flaccida, pars tensa, secondary, combination, and unclassifiable cholesteatoma were present in 20 (55.6%), 8 (22.2%), 5 (13.8%), 2 (5.6%), and case (2.8%), respectively, in the EES group and in 55 (77.5%), 8 (11.3%), 1 (1.4%), 4 (5.6%), and 3 cases (4.2%), respectively, in the MES group. The proportion of pars flaccida cases was significantly higher in the MES group compared to the EES group (*p* = 0.015). The proportion of secondary cases was significantly higher in the EES group compared to the MES group (*p* = 0.019). There was no significant difference between the two groups in pars tensa, combination, and unclassifiable cases (*p* = 0.13, *p* = 1.0, *p* = 1.0).

**Table 1:** The proportion of pars flaccida cases was significantly higher in the MES group compared to the EES group (*p* = 0.015). The proportion of secondary cases was significantly higher in the EES group compared to the MES group (*p* = 0.019). There was no significant difference between the two groups in pars tensa, combination, and unclassifiable cases (*p* = 0.13, *p* = 1.0, *p* = 1.0). EES, endoscopic ear surgery; MES, microscopic ear surgery. *, number of cases.

	EES *	MES *	*p*-Value
pars flaccida	20 (55.6%)	55 (77.5%)	0.015
pars tensa	8 (22.2%)	8 (11.3%)	0.13
secondary	5 (13.8%)	1 (1.4%)	0.019
combination	2 (5.6%)	4 (5.6%)	1.0
unclassifiable	1 (2.8%)	3 (4.2%)	1.0
total	36	71	

**Figure 3:**
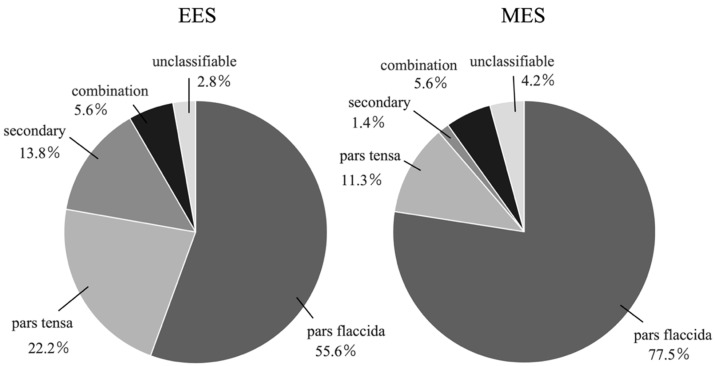
Types of cholesteatoma in the EES group and the MES group. The left chart shows the proportion of each type of cholesteatoma in the EES group, and the right chart shows the proportion in the MES group. EES, endoscopic ear surgery; MES, microscopic ear surgery.

### 3.2. Staging of Cholesteatoma (Table 2)

In the EES group, stage I, II, and III were represented in 15 (41.6%), 20 (55.6%), and 1 case (2.8%), respectively. In the MES group, those were 12 (16.9%), 55 (77.5%), and 4 cases (5.6%), respectively. The proportion of stage I cases was significantly higher in the EES group compared to the MES group (*p* = 0.0053). The proportion of stage II cases was significantly higher in the MES group compared to the EES group (*p* = 0.019). There was no significant difference between the two groups in stage III cases (*p* = 0.66).

**Table 2:** The proportion of stage I cases was significantly higher in the EES group compared to the MES group (*p* = 0.0053). The proportion of stage II cases was significantly higher in the MES group compared to the EES group (*p* = 0.019). EES, endoscopic ear surgery; MES, microscopic ear surgery. *, number of cases.

	EES *	MES *	*p*-Value
stage I	15 (41.6%)	12 (16.9%)	0.0053
stage II	20 (55.6%)	55 (77.5%)	0.019
stage III	1 (2.8%)	4 (5.6%)	0.66
total	36	71	

### 3.3. Location of Cholesteatoma (Supplementary Table S1)

The type, location, and number of cholesteatoma cases in the EES group and MES group are shown for each stage in the Supplementary Table S1.

### 3.4. Complications 

In the EES group, there was no case of injury of the chorda tympani. 

In the MES group, there were eight cases of injury of the chorda tympani (11.3%). Of these, five cases had cholesteatoma involving the nerve. The frequency of chorda tympani injury was significantly higher in the MES group (*p* = 0.049).

In the MES group, there were three cases of hematoma of the posterior ear (4.2%). All cases with hematoma were treated with bandage compression. No additional surgical treatment was required to treat hematoma. No other complications were observed in either group.

### 3.5. Recurrence of Cholesteatoma

In the EES group, one case (2.8%) of the combination type, located in the S1S2TA area, had a postoperative recurrence in the S1TA area. In the MES group, one case (1.4%) of the pars flaccida type, located in the TAM area, had a postoperative recurrence in the S2TA area. There was no significant difference between the two groups (*p* = 1.0).

### 3.6. Hearing Outcomes (Table 3A–C)

The pre- and postoperative air conduction thresholds in stages I, II, and in all cases in the EES and MES groups are shown in Table 3A,B. The postoperative improvements in air conduction thresholds for stages I, II, and in all cases in the EES and MES groups are shown in Table 3C.

In the EES group, pre- and postoperative hearing data were available for 30 cases, with 13 cases in stage I and 19 cases in stage II. In stage I cases, postoperative hearing improved significantly (*p* = 0.04), and in stage II cases and in total, there were no significant differences between preoperative and postoperative data (*p* = 0.80 and 0.44, respectively).

In the MES group, pre- and postoperative hearing data were available for 45 cases, with 4 cases in stage I, and 38 cases in stage II. No significant differences were observed between preoperative and postoperative data in stage I (*p* = 0.82), stage II (*p* = 0.14), or across all cases combined (*p* = 0.37). 

There were no significant differences in postoperative air conduction threshold improvement between the EES and MES groups in stage I (*p* = 0.25), stage II (*p* = 0.30), or overall (*p* = 0.92).

**Table 3:** (**A**) The preoperative and postoperative air conduction hearing thresholds and *p* values in stages I, II, and in total in the EES group. EES, endoscopic ear surgery. (**B**) The preoperative and postoperative air conduction hearing thresholds and *p* values in stages I, II, and in total in the MES group. MES, microscopic ear surgery. (**C**) The postoperative air conduction threshold improvements and *p* values in stages I, II, and in total in the EES group and the MES group. EES, endoscopic ear surgery; MES, microscopic ear surgery.

(**A**)			
	**Preoperative (dB)**	**Postoperative (dB)**	***p*-Value**
stage I (n = 13)	47.12 ± 21.45	40.77 ± 19.62	0.04
stage II (n =16)	40.31 ± 14.31	41.09 ± 22.45	0.80
all cases (n=30)	42.67 ± 17.95	40.96 ± 20.50	0.44
(**B**)			
	**Preoperative (dB)**	**Postoperative (dB)**	***p*-Value**
stage I (n = 4)	57.19 ± 10.28	59.69 ± 29.87	0.82
stage II (n =38)	45.49 ± 18.08	41.90 ± 17.00	0.14
all cases (n =45)	46.36 ± 18.28	44.33 ± 19.42	0.37
(**C**)			
	**EES (dB)**	**MES (dB)**	***p*-Value**
stage I (n =13 in EES, 4 in MES)	6.35 ± 9.94	−2.50 ± 21.04	0.25
stage II (n =16 in EES, 38 in MES)	−0.78 ± 12.02	3.57 ± 14.50	0.30
all cases (n =30 in EES, 45 in MES)	1.71 ± 11.97	2.02 ± 14.98	0.92

## 4. Discussion

The current study investigated the differences between MES and EES in tympanoplasty for cholesteatoma in terms of staging, complications, recurrence rates, and hearing outcomes. Notably, this is the first report to directly compare EES and MES groups by stage using the EAONO/JOS classification. Previous studies reported hearing prognosis, recurrence rates, complications, and other factors using ad hoc criteria that were not standardized. With the introduction of the world’s first standard classification, namely the EAONO/JOS, we hope that postoperative outcomes will be reported according to this standard. Understanding the extent of cholesteatoma progression using the EAONO/JOS classification is crucial for selecting the most effective treatment for the patient. Our findings revealed that EES was more commonly applied in early-stage cholesteatoma, yet the two approaches yielded no significant differences in complications, recurrence rates, or hearing outcomes.

Choice of Surgical Approach: EES was predominantly used for treating stage I cholesteatoma and secondary cholesteatoma. Previous studies have highlighted the benefits of EES for early-stage cases [17], emphasizing the usefulness of the endoscope in visualizing hidden areas [18]. Specifically, the epitympanic space and incudostapedial joint were favorable sites for endoscopic visualization [19]. Our study aligns with this, as EES was more common in early-stage pars flaccida cholesteatoma (involving the epitympanic space) and secondary cholesteatoma (often affecting the incudostapedial joint) [20,21]. These results suggest that the use of EAONO/JOS staging is useful when choosing a surgical approach.

Postoperative Complications: MES group experienced a higher tendency of complications than the EES group, including chorda tympani injury or sensorineural hearing loss. The anatomical classification of chorda tympani into five major groups [22] suggests that EES allows better identification of this structure. Previous research found no significant difference in chorda tympani injury or tases sensation between MES and EES [23]. However, our MES cases included more advanced-stage cholesteatomas, some with extracranial complications or intratemporal pathologies (classified as stage III). This case imbalance may have influenced the results. 

Recurrence rate: No significant difference in recurrence rate was observed between the two groups. In the MES group, however, some cases underwent staged surgery due to poor visual field and to avoid recurrence. We are now considering that endoscopically assisted surgery might have been better for these cases. 

Although EES has limitations in mastoid dissection compared to MES [24,25], recent advancements in instruments and techniques have improved access within mastoid cells [26]. Consistent with previous report [26], our study supports the comparable recurrence rates between EES and MES.

Postoperative Hearing Outcome: Improvement in postoperative hearing was similar between the two groups, especially for stage I and II cases. A former meta-analysis also found no significant difference in hearing outcomes between EES and MES [27]. However, stage III cases had limited EES application, preventing direct comparison due to the small sample size. Recent studies suggest EES applicability for stage III with labyrinthine fistula or with adhesive otitis media [28,29].

## 5. Limitations

First, limited patient numbers, particularly in the EES group, may have obscured significant differences in some attributes. Second, the follow-up period was relatively short, potentially underestimating recurrences. However, we performed CT scans up to one year after surgery to exclude residual cholesteatoma. Third, the choice of surgical approach varied based on individual surgeons’ preferences, reflecting real-world conditions but introducing inconsistency. Fourth, not all cases underwent MRI examinations to assess recurrence. Previous studies have reported that MRI is useful for evaluating postoperative recurrence of cholesteatoma [15,30]. We conduct postoperative CT scans at 1, 3, and 5 years after surgery for cases such as sac-type cholesteatoma, which can be completely removed as an intraoperative finding. When complete removal is uncertain, such as in cases where the cholesteatoma is extensively infiltrated, the cholesteatoma matrix ruptured during surgery, or the cholesteatoma has spread to hidden areas, we selectively use MRI to check for recurrence. Ideally, all cases would be followed up with MRI, but this is challenging due to the medical system constraints and other circumstances. This represents another limitation of this study. 

## 6. Conclusions

Early-stage cholesteatoma was treated by EES and MES. The two approaches demonstrated no difference in hearing outcomes and recurrence rates, but chorda tympani injuries occurred more frequently in MES for stages I and II. Further research is necessary for stage III.

## Data Availability

All data supporting the findings of this study are available from the corresponding author [T.Y.] on request.

## Supplementary Materials

The following supporting information can be downloaded at: https://www.mdpi.com/article/10.3390/healthcare12171737/s1, Table S1: Location of cholesteatoma by staging in the EES group and the MES group. The chart on the left has shown the location of stage I cholesteatoma, the middle chart has shown the location of stage II cholesteatoma and the chart on the right has shown the location and complications of stage III cholesteatoma in the EES and MES groups.
