# Supplementary material for: Comparison of Endoscopic and Microscopic Surgery for the Treatment of Acquired Cholesteatoma by EAONO/JOS Staging
Source: Healthcare (Basel). 2024 Aug 31;12(17):1737. doi: 10.3390/healthcare12171737 (PMC11395086; doi:10.3390/healthcare12171737)
Supplement: Supplementary file 1 [file healthcare-12-01737-s001.zip › healthcare-3138153-supplementary.pdf]

	TEES		MES	
stageI				
	space	number of cases	space	number of cases
pars flaccida	A	4	A	11
pars tensa	T	5	T	1
secondary	T	5		
unclassifiable	T	1		

	TEES		MES	
stageII				
	space	number of cases	space	number of cases
pars flaccida	S1A	6	S1A	5
	AM	5	AM	9
	TA	2	TA	5
	S1AM	1	S1AM	5
	S1S2A	1	S1TA	7
			TAM	4
			S2TA	1
			S1TAM	1
			S2TAM	2
			S1S2TA	2
			S1S2TAM	1
pars tensa	TA	2	TA	1
	S1S2TA	1	S1T	1
			S2T	1
			S1TA	1
			TAM	1
			S1TAM	2
secondary			S1A	1
combination	S1TA	1	S1TAM	1
	S1S2TA	1	S2TAM	1
unclassifiable			S1T	1
			S1AM	1
			S2TAM	1

	TEES			MES		
stageIII						
	space	number of cases	complications	space	number of cases	complications
pars flaccida	S2A	1	CW*	S1TAM	1	LF*
				AM,middle cranial fossa area	1	PB*
unclassifiable				S2TA	1	AO*
				S2TAM	1	LF

Supplementary Table S1

Location of cholesteatoma by staging in the EES group and the MES group. The chart on the left has shown the location of stage I cholesteatoma, the middle chart has shown the location of stage II cholesteatoma and the chart on the right has shown the location and complications of stage Ⅲ cholesteatoma in the EES and MES groups. The tympanomastoid space was divided into four sections; the difficult access sites (S), the tympanic cavity (T), the attic (A) and the mastoid (M). The difficult access sites (S) included S1, the supratubal recess (also called the anterior epitympanum or protympanum) and S2, the sinus tympani.EES, endoscopic ear surgery; MES, microscopic ear surgery. *LF, labyrinthine fistula; CW, canal wall destruction; AO, adhesive otitis); PB, petrous bone/skull base destruction.
